# A preliminary phylogenetic analysis of the New World Helopini (Coleoptera, Tenebrionidae, Tenebrioninae) indicates the need for profound rearrangements of the classification

**DOI:** 10.3897/zookeys.415.6882

**Published:** 2014-06-12

**Authors:** Paulina Cifuentes-Ruiz, Santiago Zaragoza-Caballero, Helga Ochoterena-Booth, Miguel Ángel Morón

**Affiliations:** 1 Departamento de Zoología, Instituto de Biología, Universidad Nacional Autónoma de México, A. P. 70-153, México, Distrito Federal. C. P. 04510; 2 Departamento de Botánica, Instituto de Biología, Universidad Nacional Autónoma de México, A. P. 70-153, México, Distrito Federal. C. P. 04510; 3 Instituto de Ecología, A.C., A. P. 63, Xalapa, Veracruz, 91000, México

**Keywords:** External morphology, Holarctic genera, Neotropical clade, Neotropical genera, male and female genitalia, polyphyly, polytomy, paraphyletic Helopini

## Abstract

Helopini is a diverse tribe in the subfamily Tenebrioninae with a worldwide distribution. The New World helopine species have not been reviewed recently and several doubts emerge regarding their generic assignment as well as the naturalness of the tribe and subordinate taxa. To assess these questions, a preliminary cladistic analysis was conducted with emphasis on sampling the genera distributed in the New World, but including representatives from other regions. The parsimony analysis includes 30 ingroup species from America, Europe and Asia of the subtribes Helopina and Cylindrinotina, plus three outgroups, and 67 morphological characters. Construction of the matrix resulted in the discovery of morphological character states not previously reported for the tribe, particularly from the genitalia of New World species. A consensus of the 12 most parsimonious trees supports the monophyly of the tribe based on a unique combination of characters, including one synapomorphy. None of the subtribes or the genera of the New World represented by more than one species (*Helops* Fabricius, *Nautes* Pascoe and *Tarpela* Bates) were recovered as monophyletic. Helopina was recovered as paraphyletic in relation to Cylindrinotina. One Nearctic species of *Helops* and one Palearctic species of *Tarpela* (subtribe Helopina) were more closely related to species of Cylindrinotina. A relatively derived clade, mainly composed by Neotropical species, was found; it includes seven species of *Tarpela*, seven species of *Nautes*, and three species of *Helops*, two Nearctic and one Neotropical. Our results reveal the need to deeply re-evaluate the current classification of the tribe and subordinated taxa, but a broader taxon sampling and further character exploration is needed in order to fully recognize monophyletic groups at different taxonomic levels (from subtribes to genera).

## Introduction

The tribe Helopini Latreille, 1802 currently contains two subtribes (Cylindrinotina and Helopina), 42 genera, and 686 species (Gebien 1943, Blackwelder 1946, Nabozhenko and Löbl 2008). A significant part of this richness is concentrated in the Palearctic Region, for which a recent catalogue is available (Nabozhenko and Löbl 2008) and where taxonomic work has been relatively constant. In contrast, only four genera are recognized for the New World, three of which are Holarctic: *Helops* Fabricius, 1775; *Tarpela* Bates, 1870; *Odocnemis* Allard, 1876, and one is exclusively Neotropical: *Nautes* Pascoe, 1876. *Odocnemis* is currently classified in the subtribe Cylindrinotina, *Helops* and *Tarpela* in the subtribe Helopina, and *Nautes* has not been classified in a subtribe because it is not included in the catalogue of Nabozhenko and Löbl (2008) as it is not present in the Palearctic region.

*Helops*, the type genus of the tribe, was described by Fabricius (1775) based on a few cephalic structures, such as the maxillary and labial palps, the labium, and the antennae of a European species, *Helops caeruleus* (Linnaeus, 1758). In the following centuries more than two hundred Palearctic species were added to this genus, but subsequent regional taxonomic revisions transferred most of them to other genera, leaving *Helops* with nine species in the region (Reitter 1922, Nabozhenko and Löbl 2008). With one exception, no such revisions have taken place for the American component of the tribe, currently composed of 150 species. In the first synoptic work for the family in North America, Horn (1870) listed 23 species of *Helops* and *Stenotrichus rufipes* LeConte, 1851, which was placed in Amphidorini, but later synonymized with *Helops* (Bouchard et al. 2005). Allard (1876, 1877), author of the only world revision of the tribe, recognized *Helops opacus* LeConte, 1859 and reassigned the remaining species among the following genera: *Diastixus* Allard, 1876 and *Coscinoptilix* Allard, 1877 with exclusively American distribution, and *Stenomax* Allard, 1876, *Nesotes* Allard, 1876 and *Catomus* Allard, 1876, with Palearctic distributions. The *Stenomax* subgenus *Omaleis* Allard, 1877, which included three species from California, was recently synonymized with *Odocnemis* Allard, 1876 by Nabozhenko (2001a). Allard included three other genera for the continent: *Hegemona* Laporte de Castelnau, 1840, *Nautes*, and *Tarpela*, which were described from Neotropical species. *Hegemona* was later transferred to Stenochiinae (Doyen 1987). Twenty-six species of *Nautes* are Neotropical (Blackwelder 1946, Papp 1961, Steiner 2006) while *Tarpela* currently contains three Nearctic species (Gebien 1943, Papp 1961), 51 Neotropical species (Blackwelder 1946) and 15 species from Asia, mainly from Japan (Nabozhenko and Löbl 2008).

In the monumental *Biologia Centrali-Americana*, Champion (1887, 1893) described approximately half of the current Helopini species known from North and Central America. Even though he was aware of the heterogeneity of the group, he synonymized Allard’s five genera with *Helops*. In his opinion, retaining Allard’s names for the species originally placed in *Helops* would have required him to propose generic names for the species in *Nautes* and *Tarpela*. Unlike *Helops*, the genera *Tarpela* and *Nautes* have more detailed taxonomic descriptions and were thought to be closely related (Bates 1870). The configuration of the prosternum and mesosternum were the main characters proposed to differentiate the two genera (Bates 1870). Champion (1887) considered these characters to be inconsistent, changing Allard’s classification by transferring two species from *Nautes* to *Helops* and *Tarpela*: *Nautes farctus* (LeConte, 1858) and *Nautes eximia* (Bates, 1870), respectively. More recently, Doyen (1988) described two Mexican species of the tribe: *Helops scintillatus* and *Helops noguerai*, but had problems assigning them to this genus because they shared characters with some species currently placed in *Nautes*.

In short, this diverse tribe includes two subtribes and multiple genera with worldwide distributions (Gebien 1943) and with different and conflicting circumscriptions, at least in the Holarctic and Neotropical components, considering from three (Champion 1887, 1893) to seven genera (Allard 1877). For the reasons detailed above, an evaluation of the recent classification seems necessary. A phylogenetic approach including all taxa is at this moment unrealistic, but a well design taxon sampling could shed light upon the naturalness of the genera and provide a basis for further research strategies aiming to translate phylogenetic hypotheses into natural classifications. The goals of this work are to explore and codify the morphological variation observed within the Neotropical helopines, for the first time test the monophyly of the subtribe Helopina and of three of the four genera present in the New World (two genera belonging in subtribe Helopina plus *Nautes* that is currently unassigned), and highlight issues in the current classification to provide guidance for future studies.

## Methods

### Phylogenetic data: taxon sampling (Table 1)

The subtribes Cylindrinotina and Helopina (Nabozhenko and Löbl 2008) were represented in the dataset by three and 20 species respectively. Taxa from three biogeographic regions were included in the sample: six Nearctic species of *Helops* *sensu* Champion (1887, 1893), one Nearctic species of *Odocnemis* *sensu* Nabozhenko (2001a), one Palearctic species from each of the following genera representing both subtribes: *Entomogonus* Solier, 1848; *Helops*, *Nalassus* Mulsant, 1854; *Probaticus* Seidlitz, 1896; *Raiboscelis* Allard, 1876; *Stenomax* Allard, 1876; *Tarpela*, and seven Neotropical species of *Nautes* and *Tarpela* according to Champion (1887, 1893). This sampling also takes into account morphological variation and tries to include all genera recognized at some point for the Neotropics. *Helops occidentalis* (Allard, 1876), *Helops sumptuosus* (Allard, 1877) and *Helops seriatus* (Allard, 1877) are not included because of lack of material. Two species of the tribe Ulomini: *Uloma mexicana* Champion, 1886 and *Hypogena biimpressa* Champion, 1886, as well as *Tenebrio molitor* Linnaeus, 1758 from the tribe Tenebrionini were incorporated as outgroups.

**Table 1.** Taxon sampling.

	Tribe	Subtribe	Species		Geographic distribution
Ingroup	Helopini	Cylindrinotina	*Nalassus plebejus*	Küster, 1850	Europe, Asia
			*Odocnemis californicus*	(Mannerheim, 1843)	Mexico, U.S.A.
			*Stenomax aeneus*	Scopoli, 1763	Europe
		Helopina	*Entomogonus peryronis*	Reiche, 1861	Asia
			*Helops aereus*	Germar, 1824	U.S.A.
			*Helops cisteloides*	Germar, 1824	U.S.A.
			*Helops farctus*	LeConte, 1858	U.S.A.
			*Helops inanis*	Allard, 1877	Mexico
			*Helops insignis*	Lucas, 1846	North of Africa
			*Helops perforatus*	Horn, 1880	Mexico, U.S.A.
			*Helops punctipennis*	LeConte, 1870	U.S.A.
			*Helops rossii*	Germar, 1817	Europe
			*Helops rufipes*	(LeConte, 1851)	Mexico, U.S.A.
			*Probaticus tentyrioides*	Küster, 1851	Asia, Europe
			*Raiboscelis corvinus*	Küster, 1850	Asia, Europe
			*Tarpela aerifera*	Allard, 1876	Mexico, Central America
			*Tarpela browni*	Bates, 1870	Nicaragua
			*Tarpela contigua*	Champion, 1887	Mexico
			*Tarpela cordicollis*	Marseul, 1876	Japan
			*Tarpela costata*	Champion, 1887	Mexico, Nicaragua
			*Tarpela depressa*	Champion, 1887	Mexico
			*Tarpela reticulata*	Champion, 1887	Honduras
			*Tarpela torrida*	Champion, 1887	Mexico
		unassigned	*Nautes belti*	Allard, 1877	Central America
			*Nautes enoplopoides*	Champion, 1887	Guatemala
			*Nautes fervidus*	Pascoe, 1866	Mexico, Central America
			*Nautes magnificus*	Champion, 1887	Guatemala
			*Nautes splendens*	Champion, 1887	Panama
			*Nautes striatipennis*	Champion, 1887	Mexico
			*Nautes varians*	Champion, 1887	Mexico
Outgroup	Ulomini		*Uloma mexicana*	Champion, 1886	Mexico, Central America
			*Hypogena biimpressa*	Champion, 1886	Mexico, Central America, South America
	Tenebrionini		*Tenebrio molitor*	Linnaeus, 1758	global

Specimens were kindly loaned by curators at the following national and international institutions:

AMNH American Museum of Natural History, New York, NY, USA (Lee Herman)

BNHM The Natural History Museum, London, U. K. (Max Barclay)

CASC California Academy of Sciences, San Francisco, CA, USA (Dave Kavanaugh)

CNIN Colección Nacional de Insectos, Intituto de Biología, UNAM, Mexico City, Mexico (Santiago Zaragoza Caballero)

EMEC Essig Museum of Entomology, University of California, Berkeley, CA, USA (Peter T. Oboyski)

FMNH Field Museum of Natural History, Chicago, IL, USA (James Boone)

HNHM Hungarian Natural History Museum, Budapest, Hungary (Otto Merkl)

IEXA Colección entomológica, Instituto de Ecología, A. C., Xalapa, Veracruz, Mexico (Miguel Ángel Morón & Delfino Hernández)

MNHN Museum National d’Histoire Naturelle, Paris, France (Antoine Mantilleri)

LACM Natural History Museum of Los Angeles County, Los Angeles, CA, USA (Weiping Xie)

NMNH National Museum of Natural History, Smithsonian Institution, Washington, DC, USA (Warren Steiner & David Furth)

OSUC C. A. Triplehorn Insect Collection, Ohio State University, Columbus, OH, USA (Charles A. Triplehorn & Luciana Musetti)

SBMNH Santa Barbara Museum of Natural History, Santa Barbara, CA, USA (Michael Caterino)

TAMU Texas A & M University Insect Collection, College Station, TX, USA (Edward Riley)

UCDC Bohart Museum, University of California, Davis, CA, USA (Steve Heydon)

ZMHB Museum für Naturkunde der Humboldt-Universitat, Berlin, Germany (Bernd Jaeger)

### Phylogenetic data: characters

Two hundred eighty-one specimens were examined with an Olympus SZH10 stereomicroscope (magnification: 17.5–350×) equipped with an ocular graticule for length measurements, and a drawing tube. Morphological characters were measured as follows: width of the head was measured across the vertex, length of the last antennomere in the female was measured along its longest edge; width was measured across its widest point; length of pronotum was measured along the midline from its anterior edge to its posterior edge; width was measured across its widest point. Puncture density follows modified conventions used by Paulsen (2005) and Smith et al. (2011): either confluent (separated by one or less than a puncture diameter), moderate (separated by 2–3 puncture diameters), or sparse (separated by 4 or more puncture diameters). Nomenclature and interpretation of female genital tract follows Tschinkel and Doyen (1980) and Doyen (1994).

**Figure 1.**
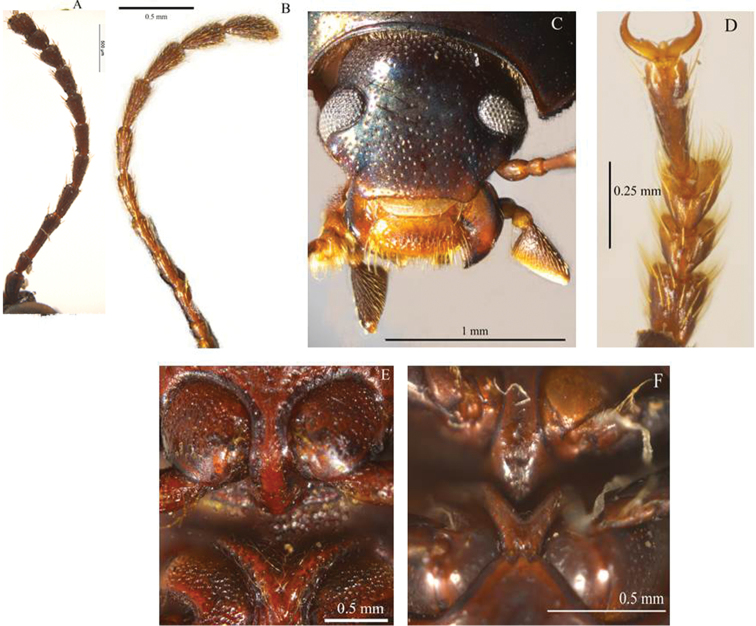
Examples of external characters (mainly diagnostic), traditionally used in *Helops*, *Tarpela* and *Nautes*, (number of character: character state): **A** broad apical antennomere (5:0), shorter than the third antennomere (4:0) illustrated from *Helops aereus* Germar **B** filiform apical antennomeres (5:2), as long as the third antennomere (4:1) illustrated from *Nautes fervidus* Pascoe **C** male maxillary palps with length of inner edge 2.6–2.9 times the length of posterior edge (7:2) illustrated from *Nautes chrysomeloides* Champion **D** third lobate segment of male tarsi (30:0) and short fourth tarsomere (31:0) illustrated from *Nautes fervidus* **E** not prominent prosternum (27:1) in *Helops cisteloides* Germar **F** prominent-acute prosternum (27:0) in *Nautes fervidus*.

**Figure 2.**
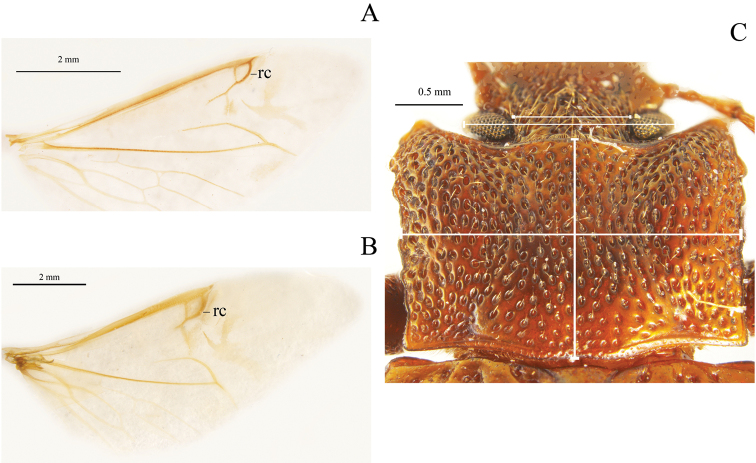
Examples of non-traditional external characters in Helopini: **A** reduced recurrent cell (rc) (26:0) in fully developed wing (25:1) of *Helops californicus* Mannerheim **B** wide recurrent cell (rc) (26:1) in fully developed wing (25:1) of *Tarpela aerifera* Allard **C** head width and interocular width (6:0) and pronotum width and length (18:0) in *Tarpela costata* Champion 1887, showing a gibbous pronotum disk surface (8:0), with very dense (9:0) and very deep (10:0) pronotum punctures.

**Figure 3.**
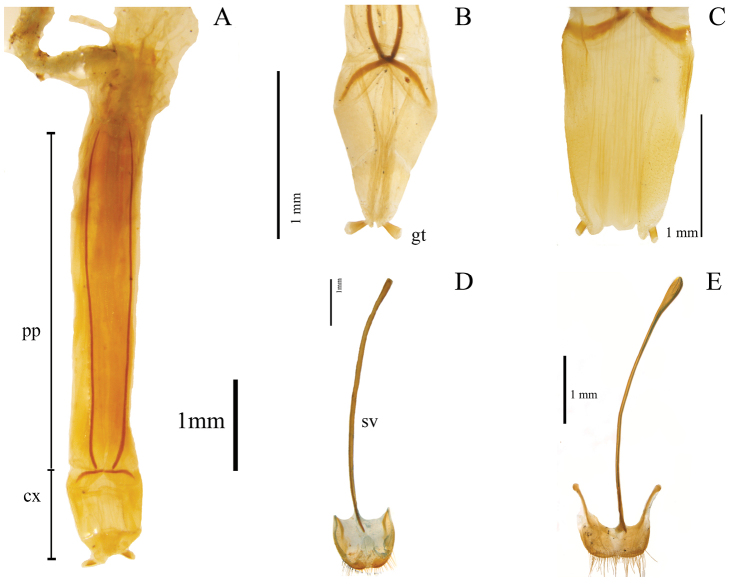
Internal morphological characters (female genitalia) in Helopini: **A** length of paraproct (pp) three or more times length of coxite (cx) (39:0) illustrated from the ovipositor of *Helops cisteloides* Germar **B** long gonostyles (gt) (37:1) with wide apex (38:1), represented by the ovipositor of *Odocnemis exaratus* Germar, not included in the analysis **C** reduced gonostyles (37:0), with base as wide as apex (38:0) represented by the ovipositor of *Tarpela micans* (Fabricius), not included in the analysis **D** blunt, narrow apex of eighth sternite (33:0), not evident arms (34:1) and not dilated distal end of the spiculum ventrale (sv) (35:1) illustrated from *Helops cisteloides* **E** trapeziform apex of eighth sternite (33:1), evident arms (34:0) and dilated distal end of spiculum ventrale (35:0), represented by sclerite of *Odocnemis exaratus*.

**Figure 4.**
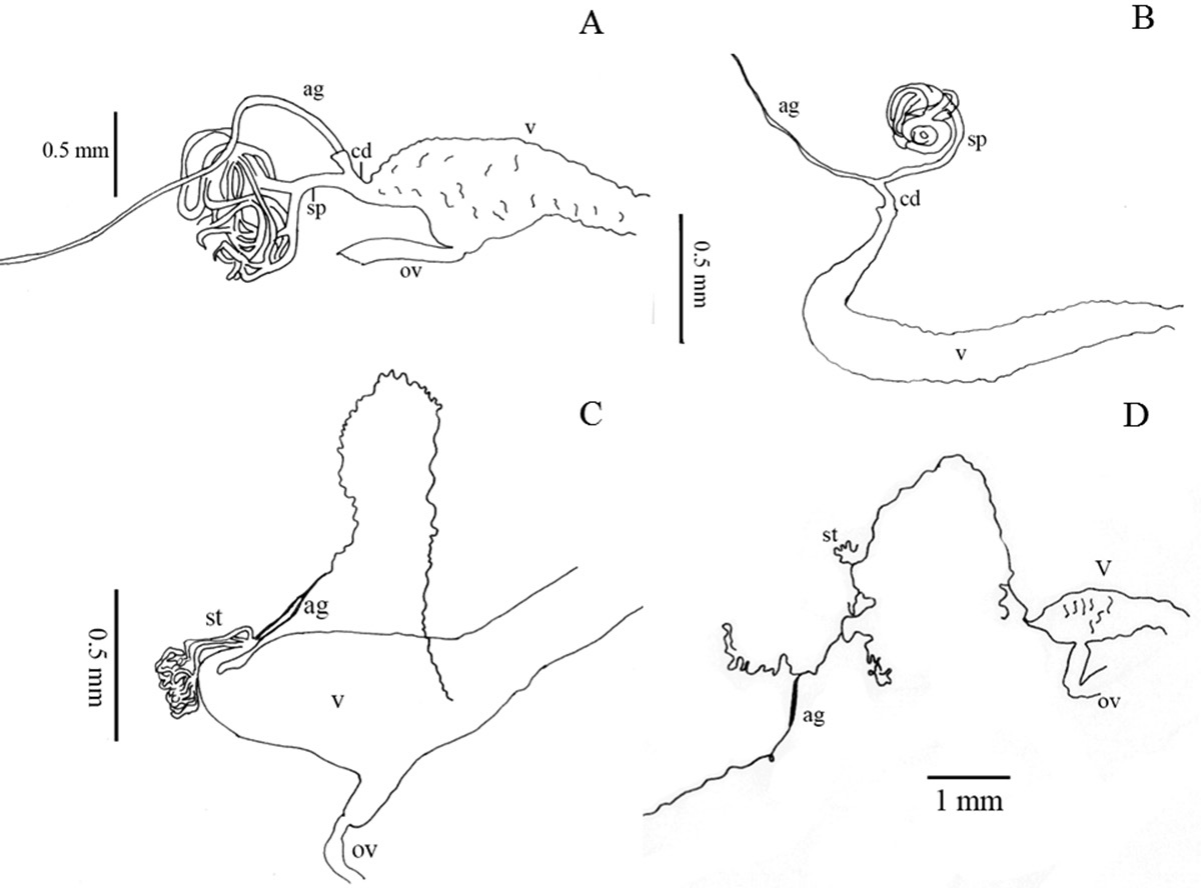
Internal morphological characters (female genitalia) representing the different morphological types found in our sample of Helopini ag = accessory gland, sp = spermatheca, st = spermathecal tube(s), cd = common duct of accessory gland and spermatheca, v = vagina, ov = oviduct: **A** infundibular vagina (40:0), single spermatheca branched near its base (41:0, 42:0) and accessory gland in the common duct (49:1) illustrated from *Helops insignis* Germar representing the helopiod type (Nabozhenko 2001b, 2002a, 2002b, 2005) **B** vagina strongly curved and narrowed before the apex (40:1), single spermatheca not branched near the base (41:0, 42:1) illustrated from *Nalassus plebejus* Küster representing the nalassoid type (Nabozhenko 2001b, 2002a, 2002b) **C** female genital tract with three serial spermathecal tubes (41:1) close to each other (43:0) and terminal accessory gland (49:2) in *Helops farctus* LeConte, illustrating the pattern previously reported for some Pimeliinae species (Doyen 1994), here reported for the first time in Tenebrioninae **D** distant spermathecal tubes (43:1) in *Helops perforatus* Horn with terminal accessory gland (49:2), illustrating a pattern described here for the first time. Total length of the accessory gland is not represented in **A** and **B.**

**Figure 5.**
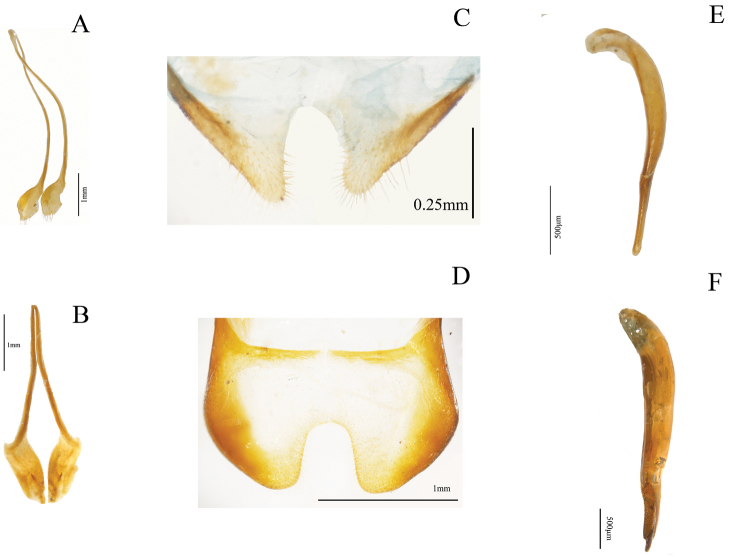
Internal morphological characters (male genitalia) representing the different morphological types found in our sample of Helopini: **A** pleural rods of gastral spicula close only at the end (50:2), representing the nalassoid type (Nabozhenko 2001b, 2002a), illustrated from *Stenomax aeneus* (Scopoli) **B** pleural rods of gastral spicula close towards the middle of their length (50:0), representing the helopiod type (Nabozhenko 2001b, 2002a, 2005), illustrated from *Tarpela micans* (Fabricius), not included in the analysis **C** narrow and acute lobes of eighth sternite (54:1) and deep notch (52:0) in *Helops farctus* LeConte **D** broad lobes of eighth sternite (54:0) and shallow notch (52:1) in *Stenomax aeneus* **E** projected anterior part of basal piece (basal piece “J” shaped) in *Odocnemis californicus* Mannerheim (67:0) **F** anterior part of basal piece not projected in *Nautes fervidus* Pascoe (67:1), character state used for the first time in this study.

**Figure 6.**
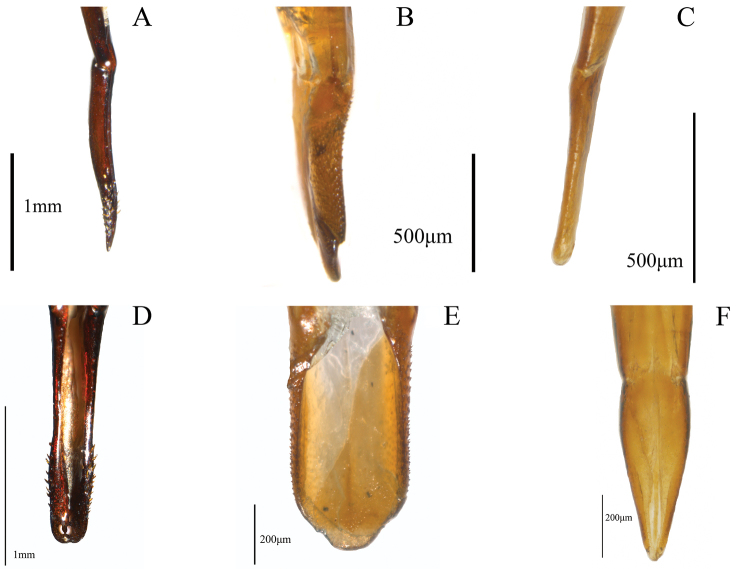
Aedeagal characters (male genitalia) representing the different morphological types found in or sample of Helopini: **A** evident setae (57: 0) representing the helopiod type (Nabozhenko 2001b, 2002a, 2005), distributed over half of the parameres (58:0), illustrated from *Helops caeruleus* (Linnaeus), not included in the analysis (lateral view) **B** evident setae (57:0); representing the catomoid type (Nabozhenko 2006), distributed over two thirds of parameres (58:1) in *Nautes fervidus* Pascoe **C** not evident setae over parameres (57:1) representing the nalassoid type (Nabozhenko 2001b, 2002a, 2002b), illustrated from *Odocnemis californicus* Mannerheim **D** parameres not convergent (59:1), with blunt apex (62:0) in *Helops caeruleus* (ventral view) **E** parameres not convergent (59:1) with weakly constricted apex (61:1) in *Nautes fervidus* **F** parameres convergent (59:0), with acute (60:1) not constricted apex (62:1) in O. *californicus*.

Thirty-two characters correspond to external morphology; characters used in generic descriptions (Pascoe 1866, Bates 1870) or in previous phylogenetic studies (Doyen and Tschinkel 1982) were included (Figs 1, 2). The remaining 35 are based on male and female genitalia. Internal characters (Figs 3–6) were coded according to previous works (Antoine 1947, Doyen 1994, Flores 1996, Nabozhenko 2001a, Aalbu 2005, Rosas et al. 2011) independent of the fact that some were used to investigate other families as they are considered to be useful in Tenebrionidae as well (Rosas et al. 2011). Two characters (35, 43) plus two character states (67: 1, 2) were used for the first time. Female genitalia were dissected, cleared and stained following Tschinkel and Doyen (1980), replacing NaOH with KOH. Photographs were taken using a Leica microscope equipped with a camera Leica Z16 APO A. The imaging software used was Leica Application Suite 2.8.1.

In total, 44 binary and 23 multistate characters were coded and treated as non-additive. Individual consistency and retention indices (ci, ri) are provided for all characters from the consensus tree (synapomorphies have a value of 1 for both indices). Four additional characters (listed and explained at the end of the character list) were initially explored but removed from the final analysis due to their high homoplasy, assessed by a character removal methodology (see below).

1. Shape of union between clypeus and frons: (0) clypeus slightly depressed, weak fronto-clypeal suture (Fig. 1C); (1) clypeus strongly depressed, evident fronto-clypeal suture (ci = 0.25; ri = 0.80).
2. Length of antennae (male): (0) short, slightly over posterior margin of pronotum; (1) long, clearly surpassing posterior margin of pronotum (ci = 0.33; ri = 0.50).
3. Shape of antennae: (0) filiform (Fig. 1B); (1) submoniliform (synapomorphy).
4. Length of third antennomere: (0) longer than apical antennomeres (Fig. 1A); (1) shorter than or as long as apical antennomeres (Fig. 1B) (ci = 0.14; ri = 0.57).
5. Size of last antennomere (female): (0) almost as long as wide or wider than long (Fig. 1A); (1) 2.5 or more times as long as wide; (2) 1.5-2 times as long as wide (Fig. 1B) (ci = 0.30; ri = 0.30).
6. Head width (Fig. 2C): (0) 1.5 times width of intraocular space; (1) less than 1.5 times width of intraocular space (ci = 0.25; ri = 0).
7. Length of inner edge of maxillary palp (male): (0) 1-1.5 times length of posterior edge; (1) 1.6-2.5 times length of posterior edge; (2) 2.6-2.9 times length of posterior edge (Fig. 1C) (ci = 0.18; ri = 0.25).
8. Pronotum disk surface: (0) gibbous (Fig. 2C); (1) not gibbous (ci = 0.33; ri = 0.50).
9. Density of pronotum punctures: (0) very dense or confluent; (1) moderately dense; (2) sparse (ci = 0.25; ri = 0.40).
10. Depth of pronotum punctation: (0) deep (more than 20 μm) (Fig. 2C); (1) medium (10-20 μm); (2) shallow (less than 10 μm) (ci = 0.25; ri = 0.64).
11. Setae of head and pronotum (observed at a magnification up to 140X): (0) present; (1) not evident (ci = 0.50; ri = 0.60).
12. Width of lateral carinae of pronotum: (0) lateral carinae 2-5 times width of anterior carinae; (1) lateral carinae less than 2 times width of anterior carina (ci = 0.33; ri = 0.50).
13. Shape of anterior angles of pronotum: (0) acute; (1) blunt or slightly acute; (2) straight (ci = 0.25; ri = 0.40).
14. Lateral sides of pronotum: (0) with crenate carinae; (1) with smooth carinae; (2) without conspicuous carinae (ci = 0.40; ri = 0.40).
15. Shape of posterior angles of pronotum: (0) straight; (1) acute; (2) blunt; (3) obtuse (ci = 0.40; ri = 0.40).
16. Projection of posterior angles of pronotum: (0) strong; (1) weak (Fig. 2C) (ci = 0.50; ri = 0.50).
17. Posterior margin of pronotum: (0) convex; (1) straight; (2) bisinuate (Fig. 2C) (ci = 0.40; ri = 0.66).
18. Pronotum shape: (0) rectangular (its width 1.5 times or more its length) (Fig. 2C); (1) almost square (its width less than 1.5 its length) (ci = 0.50; ri = 0.92).
19. Propleura texture: (0) strongly rugose or punctated; (1) smooth or slightly rugose or punctated (ci = 0.20; ri = 0.33).
20. Elytra shape in lateral view: (0) strongly arcuate; (1) slightly arcuate; (2) more evident towards the middle and posteriorly (ci = 0.33; ri = 0.42).
21. Pronotum tegument: (0) smooth; (1) chagrined (ci = 0.16; ri = 0.28).
22. Elytra punctures: (0) in grooves; (1) in rows (ci = 0.12; ri = 0.50).
23. Shape of elytral interstriae: (0) convex; (1) flat; (2) acute (ci = 0.16; ri = 0.09).
24. Elytral tegument: (0) lustrous; (1) dull (ci = 0.14; ri = 0.33).
25. Metathoracic wings: (0) brachypterous or not evident; (1) fully developed (Figs 2A–B) (ci = 0.11; ri = 0.38).
26. Size of recurrent cell: (0) reduced (due to the approximation of the radial cross-vein to the recurrent radius) (Fig. 2A); (1) wide (due to the separation of the radial cross-vein to the recurrent radius) (Fig. 2B) (ci = 0.50; ri = 0.75).
27. Shape of prosternal process apex in ventral view: (0) strongly projected (Fig. 1F); (1) weakly, or not projected (Fig. 1E) (ci = 0.25; ri = 0.40).
28. Shape of prosternal process apex in lateral view: (0) straight; (1) declivous (ci = 0.14; ri = 0.25).
29. Density of leg punctures: (0) femur punctures sparser than tibia punctures; (1) density of femur and tibia punctures similar (ci = 0.14; ri = 0.53).
30. Shape of third tarsomere: (0) lobate (Fig. 1D); (1) not lobate (ci = 0.16; ri = 0.54).
31. Size of fourth tarsomere: (0) shorter than third tarsomere (Fig. 1D); (1) as long as third tarsomere (ci = 0.20; ri = 0.66).
32. Density of punctures and pubescence of abdominal ventrites (male): (0) high towards middle of ventrites 1-3; (1) homogeneous on ventrites 1-5; (2) high towards middle of ventrites 1-5 (ci = 0.15; ri = 0.42).
33. Shape of inner sternite VIII (female): (0) blunt and narrow (Fig. 3D); (1) trapeziform or blunt and wide (Fig. 3E) (ci = 0.33; ri = 0).
34. Arms of spiculum ventrale (female): (0) evident (Fig. 3E); (1) not evident (Fig. 3D) (ci = 0.20; ri = 0.33).
35. Shape of distal end of stalk of spiculum ventrale (female): (0) round or oval and dilated (Fig. 3E); (1) round but not dilated (Fig. 3D) (ci = 0.50; ri = 0.80).
36. Length/width ratio of gonostyles (female): (0) length twice or more its width; (1) length less than twice its width (ci = 0.25; ri = 0.25).
37. Relative length of coxites (female): (0) 8 or more times gonostyle length (Fig. 3C); (1) less than 8 times gonostyle length (Fig. 3B) (ci = 0.33; ri = 0.33).
38. Shape of gonostyles (female): (0) apex as wide as base (Fig. 3C); (1) with apex wider than base (Fig. 3B) (ci = 0.25; ri = 0.62).
39. Relative length of paraproct (female): (0) three or more times coxite length (Fig. 3A); (1) two times coxite length; (2) as long as coxites; (3) less than coxite length (ci = 0.37; ri = 0.50).
40. Shape of vagina (female): (0) infundibular or sacciform, curved or not at the apex (i.e., at the connection with the spermatheca or common duct) (Fig. 4A–C); (1) sacciform and strongly narrowed and curved before the apex (Fig. 4B) (synapomorphy).
41. Number of spermathecal tubes: (0) one (Fig. 4A-B); (1) more than one (Fig. 4C–D) (synapomorphy).
42. Spermathecal tubes structure: (0) branched near the base (Fig. 4A); (1) not branched, branched at the base (looking like a fascicule of tubes), or branched far from the base (Fig. 4B–D) (ci = 0.33; ri = 0.77).
43. Spermathecal tubes arrangement: (0) near to each other (Fig. 4C); (1) distant from each other (Fig. 4D) (ci = 0.50; ri = 0.50).
44. Common duct: (0) present; (1) absent (ci = 0.16; ri = 0).
45. Length of common duct of spermatheca and accessory gland: (0) long (Fig. 4D); (1) short (Fig. 4C); (2) intermediate (Fig. 4B) (ci = 0.20; ri = 0.27).
46. Position of common duct (female): (0) apical to vagina (Fig. 4A-D); (1) anterior to vagina apex (ci = 0.33; ri = 0).
47. Width of spermathecal tube(s) (female): (0) increases distally; (1) homogeneous width or gradually decreasing (ci = 0.50; ri = 0.66).
48. Texture of spermathecal tubes: (0) smooth; (1) annulate (synapomorphy).
49. Position of accessory gland: (0) emerging directly from the vagina, far from the spermatheca; (1) in the common duct (Fig. 4A-B); (2) terminal to the spermathecal tubes and common duct (Fig. 4C-D) (synapomorphy).
50. Arrangement of pleural rods of gastral spicula (male): (0) close towards the middle of their length (Fig. 5B); (1) close towards the proximal third; (2) close only at the end (Fig. 5A) (ci = 0.50; ri = 0.66).
51. Shape of pleural rods of gastral spicula (male): (0) straight of slightly curved (Fig. 5B); (1) strongly curved (Fig. 5A) (ci = 0.33; ri = 0.33).
52. Depth of notch of eighth sternite (male) measured as the ratio of sternite length (SL) and notch length (NL): (0) deep (SL/NL <3) (Fig. 5C); (1) shallow (SL/NL >3) (Fig. 5D); (2) without notch (ci = 0.50; ri = 0.81).
53. Width of notch of eighth sternite (male): (0) wide; (1) narrow (ci = 0.33; ri = 0).
54. Shape of lobes of eighth sternite (male): (0) notably and anteriorly wide (Fig. 5D); (1) narrow and acute or slightly blunt (Fig. 5C) (ci = 0.20; ri = 0.66).
55. Relative length of basal piece (male): (0) three or more times the length of parameres; (1) less than three times the length of parameres (Fig. 5E–F) (ci = 0.16; ri = 0).
56. Shape of parameres in lateral view (male): (0) sinuate (Fig. 6A); (1) straight or slightly curved (Fig. 6B-C) (ci = 0.25; ri = 0.57).
57. Setae on parameres (male): (0) present (Fig. 6D-E); (1) not evident (observed at a magnification up to 140X) (Fig. 6F) (ci = 0.25; ri = 0.66).
58. Distribution of evident setae on parameres (male): (0) covering apical half of parameres (Fig. 6D); (1) covering more than two thirds of parameres (Fig. 6E) (synapomorphy).
59. Sides of parameres in ventral view (male): (0) convergent to the apex, with a fusiform space in between (Fig. 6F); (1) not convergent (Fig. 6D–E) (ci = 0.50; ri = 0.92).
60. Constriction of the apex of parameres (male): (0) present (Fig. 6E); (1) absent (ci = 0.25; ri = 0.70).
61. Constriction of the apex of parameres (male): (0) strong; (1) weak (Fig. 6E) (ci = 0.33; ri = 0).
62. Shape of the apex of parameres (male): (0) blunt or straight (Fig. 6D); (1) acute (Fig. 6F); (2) fan shaped (ci = 0.50; ri = 0.80).
63. Apical projection of parameres in ventral direction (lateral and ventral view): (0) present; (1) absent (ci = 0.33; ri = 0.33).
64. Apical compression of parameres view laterally as a dorsal or dorso-ventral projection or keel: (0) present; (1) not evident (observed at a magnification up to 140X) (synapomorphy).
65. Width of parameres (male) at the middle: (0) narrower than basal piece (Fig. 6D); (1) as wide as basal piece (Fig. 6E–F) (ci = 0.25; ri = 0.57).
66. Shape of apex of median lobe (male): (0) blunt or with an inconspicuous notch; (1) lobate; (2) constricted (ci = 0.33; ri = 0.73).
67. Shape of anterior part of basal piece (male): (0) projected, basal piece “J” shaped (Fig. 5E); (1) not projected (Fig. 5F); (2) projected, basal piece “C” shaped (ci = 0.66; ri = 0.92).

**Removed characters:**

1. Width of pronotum: (0) widest towards the middle; (1) widest before middle; (2) widest at posterior margin or from middle to posterior margin.
2. Length of pronotum setae: (0) long (more than 100 μm); (1) short (less than 50 μm).
3. Projection of anterior angles of pronotum: (0) strong; (1) weak or absent.
4. Diameter of elytra punctures: (0) reduced (less than 200 μm); (1) large (more than 200 μm).

### Phylogenetic analysis

The matrix was compiled using WinClada (Nixon 2002). Heuristic searches were conducted through NONA (Goloboff 1999) with multiple Tree Bisection and Reconnection (TBR) using 1,000 initial Wagner trees (mult*1000), holding 20 trees per replication (hold/20) and expanding the memory for a final TBR to completion with up to 10,000 trees (max*10000). The cladograms were rooted with *Uloma mexicana*. All most parsimonious trees (MPTs) found were collected, and ambiguously supported branches were collapsed in WinClada. Identical trees were then removed and a consensus was calculated using the option “Strict” in WinClada.

A simple sequential character removal analysis (modified after Davis et al. 1993) was carried out as implemented in WinClada (Nixon 2002), using the same search parameters as explained. The length of the resulting 71 consensus trees (one for each matrix resulting from the progressive removal of the 71 characters) was compared to determine the influence of each character in the topology of the consensus of the MPTs. In this way, four characters (listed above) were detected to particularly introduce conflict in the analysis due to high homoplasy values and were removed from the matrix. When removing each of these characters, the length of the consensus decreased by more than 30 steps and the resolution of the topology greatly improved. The final 67-character matrix (character listed and explained above) was then analyzed with the parameters described in the previous paragraph. These characters are mapped onto the consensus only if their optimization was not ambiguous and if they were present among all the MPTs. This was assessed using the option “Map Common synapomorphies” on the sub-menu “Synapomophies” menu “Optimize” of TNT (Goloboff et al. 2003). The consensus was used to map homoplasy at the level of characters in WinClada; a metafile was created and the tree was edited using Corel Draw X6 (Corel Corporation 2012).

To evaluate statistical branch support, a bootstrap analysis was conducted with NONA (Goloboff 1999) through WinClada (Nixon 2002). For this analysis 1,000 replicates were conducted for each using 100 initial trees holding 20 trees and expanding the memory up to 1,000 trees (mult*100 hold/20 max*1000). Frequencies were calculated on the consensus of the 67-character matrix and only values above 50% are shown.

## Results

**Figure 7.**
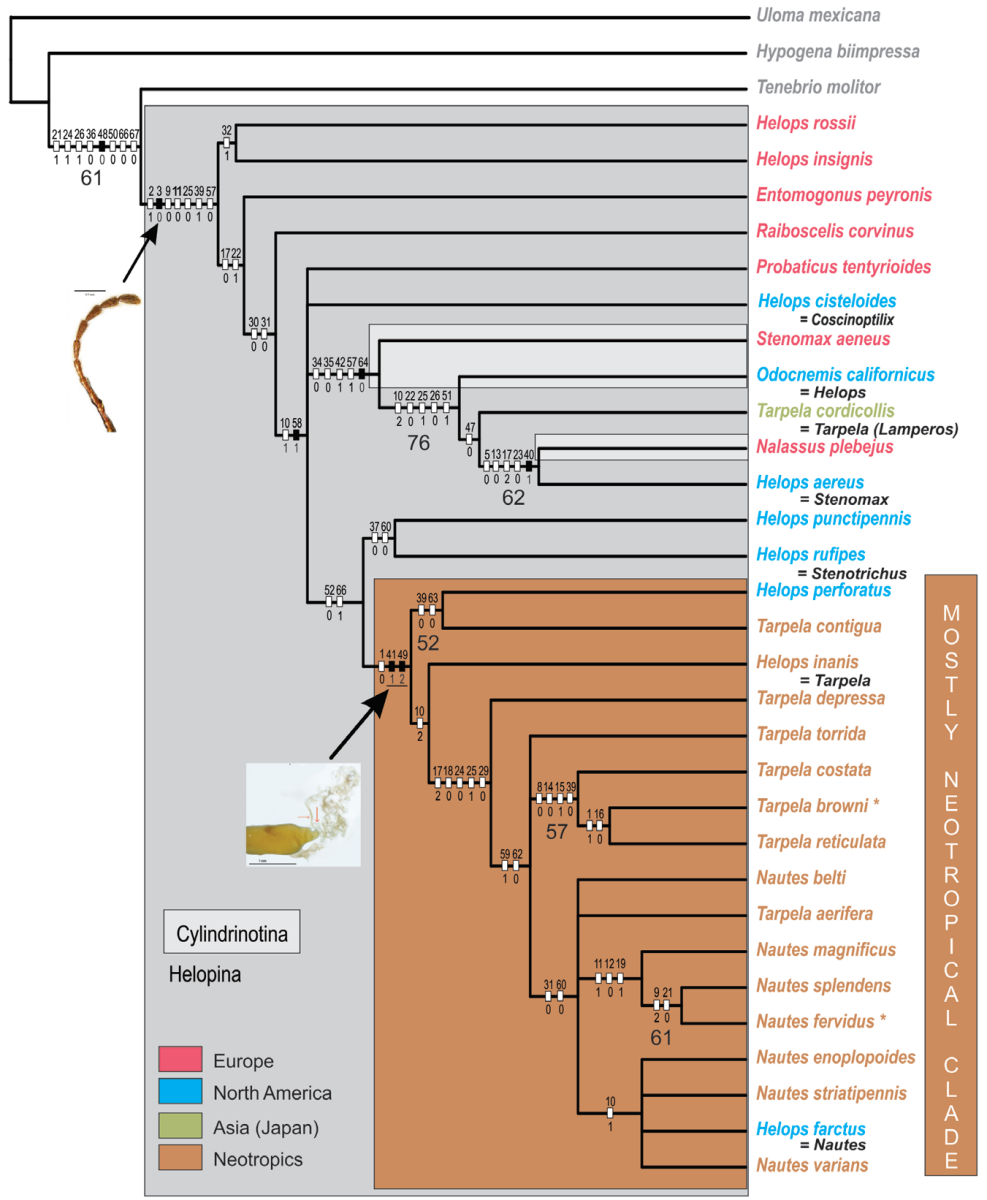
Strict consensus of 12 most parsimonious trees (L = 314; ci = 0.28; ri = 0.56). Characters are mapped onto the consensus only if their optimization is not ambiguous and if they are present among all the MPTs. The consensus is used to map homoplasy at the level of characters. Black rectangles represent single, non-homoplasious character state transformations, and white rectangles represent multiple, homoplasious character state transformations. The number depicted above each rectangle represents the character and the number below the rectangle represents the character state. The bigger number below the branches corresponds to Bootstrap values over 50%. The combination of characters for each terminal is not shown. Three important synapomorphies are illustrated in the cladogram: the filiform antennae (3:0), as the single synapomorphy of the tribe, and the number of spermathecal tubes (41:1) plus the terminal position of the accessory gland (49:2), as the synapomorphies supporting a mostly Neotropical clade. These character states are reported for the first time for the tribe. Two shades of gray in the cladogram indicate the subtribe to which the terminals belong (except *Nautes*). Colors in the terminals indicate their geographic distribution. Below seven terminals the former classification (genus or subgenus) is shown. An asterisk indicates the type species included in the analysis: *Tarpela browni* and *Nautes fervidus*.

The 67-character matrix (Table 2) yielded 12 most parsimonious trees with 301 steps (length = L), a consistency index (ci) of 0.29, and a retention index (ri) of 0.59. The strict consensus (L = 314; ci = 0.28; ri = 0.56) is presented in Figure 7. Six out of seven characters retrieved as synapomorphies are from internal morphology. Four synapomorphies correspond to the female genitalia: vagina strongly curved in the apex (character 40: state 1), more than one spermathecal tube (41:1), smooth texture of spermathecal tube (48:0), and terminal position of the accessory gland (49:2). Two synapomorphies correspond to the male genitalia: distribution of evident setae on the parameres (58:1), and presence of a dorsal projection or keel on the parameres (64:0). One synapomorphy corresponds to external morphology: the filiform shape of the antennae: (3:0). Although only six clades had bootstrap values over 50%, most clades are supported by a unique combination of at least two characters.

**Table 2.** Matrix for the cladistics analysis of the tribe Helopini (Coleoptera, Tenebrioninae, Tenebrionini); “-” represents inapplicable character states, “?” represents not observed data.

Taxon/character					5					10					15					20					25					30					35					40					45					50					55					60					65		
*Uloma mexicana*	1	0	1	1	0	0	0	1	1	0	1	1	1	1	0	1	2	1	0	1	0	0	0	0	1	0	1	1	0	1	1	1	1	0	?	1	0	0	3	0	0	0	-	0	1	1	0	1	1	2	0	2	-	-	0	0	1	-	0	0	1	2	1	1	1	2	2
*Helops punctipennis*	1	1	0	1	3	0	1	1	0	2	0	1	1	1	0	1	2	1	0	1	1	0	1	1	1	1	1	1	1	1	0	1	1	1	1	1	0	0	2	0	0	0	-	0	2	0	1	0	1	0	0	0	0	0	0	1	0	1	0	0	0	1	0	1	1	1	1
*Entomogonus peyronis*	1	1	0	0	0	0	?	1	0	0	0	1	1	2	0	1	0	1	0	1	1	1	1	1	0	-	1	1	1	1	1	2	1	1	1	0	1	0	1	0	0	0	-	0	2	0	1	0	1	0	0	?	?	?	1	0	0	0	0	1	-	0	1	1	0	?	0
*Raiboscelis corvinus*	0	1	0	0	3	0	1	1	0	0	0	1	1	1	3	1	0	1	0	1	1	1	1	1	0	-	1	1	1	0	0	2	?	1	1	0	1	0	1	0	0	0	-	0	1	0	1	0	1	0	0	1	0	0	1	0	0	0	0	1	-	0	1	1	0	0	0
*Probaticus tentyrioides*	1	1	0	0	2	0	2	1	0	1	0	1	1	1	3	1	0	1	0	0	1	-	-	1	0	-	1	1	0	0	0	1	1	1	1	0	1	1	1	0	0	0	-	0	1	0	1	0	1	0	0	1	0	0	1	1	0	1	0	1	-	0	1	1	0	0	0
*Helops rossii*	1	1	0	0	0	0	0	1	0	0	0	1	1	1	0	1	2	1	0	1	1	0	1	0	0	-	1	1	1	1	1	1	1	1	1	0	1	0	1	0	0	0	-	0	1	0	1	0	1	0	0	1	0	0	1	0	0	0	0	1	-	0	1	1	0	0	0
*Helops insignis*	1	1	0	0	3	0	0	1	0	0	0	1	1	1	0	1	2	1	0	1	1	0	1	1	0	-	1	1	1	1	1	1	1	1	1	0	1	0	1	0	0	0	-	0	1	0	1	0	1	0	0	1	0	0	1	0	0	0	0	1	-	0	1	1	0	0	0
*Helops cisteloides*	1	1	0	0	2	0	1	1	0	1	0	1	1	1	0	1	0	1	0	1	1	1	1	0	0	-	1	1	0	0	0	2	0	1	1	1	1	0	0	0	0	0	-	0	2	0	1	0	1	0	0	1	0	1	0	0	0	1	0	1	-	0	1	1	0	0	0
*Nautes enoplopoides*	0	1	0	1	?	0	1	1	2	-	0	1	2	1	0	1	2	0	1	0	?	0	1	1	0	-	1	0	1	0	0	2	1	1	1	0	1	1	0	0	1	1	0	1	-	-	1	0	2	0	0	0	1	0	1	1	0	1	1	1	-	0	1	1	1	1	1
*Helops perforatus*	0	1	0	0	2	0	1	1	0	1	0	1	1	1	0	1	1	1	0	1	1	1	1	0	0	1	1	1	1	1	1	2	1	1	1	0	1	1	0	0	1	1	1	0	0	0	1	0	2	1	0	0	0	0	1	1	0	1	0	1	-	1	0	1	1	1	1
*Nautes striatipennis*	0	1	0	1	1	0	1	1	0	1	0	1	0	1	0	1	2	0	0	1	1	0	1	0	1	1	0	0	0	0	0	2	1	1	1	0	1	1	0	0	1	1	0	0	1	0	1	0	2	0	0	0	0	1	1	1	0	1	1	0	1	0	1	1	1	1	1
*Helops farctus*	0	1	0	1	2	1	1	1	0	1	0	1	0	1	0	1	2	0	0	0	1	0	0	0	0	-	1	1	0	0	0	0	1	1	1	0	1	1	1	0	1	1	0	0	1	0	1	0	2	0	0	0	0	1	1	1	0	1	1	0	0	0	1	1	1	1	1
*Nautes splendens*	0	1	0	1	2	0	2	1	2	2	1	0	0	1	0	1	2	0	1	0	0	1	1	0	1	1	0	0	0	0	0	0	1	1	1	?	?	?	1	0	1	1	0	0	1	0	1	0	2	0	0	0	0	1	1	1	0	1	1	0	1	0	1	1	1	1	1
*Tarpela browni*	1	1	0	1	?	0	1	0	0	0	0	1	0	0	1	0	2	0	0	1	?	1	1	0	1	1	1	1	0	0	1	0	1	1	1	0	1	1	0	0	1	1	0	0	1	0	1	0	2	0	0	0	0	0	1	1	0	1	1	1	-	0	1	1	1	0	1
*Tarpela torrida*	0	1	0	1	2	0	2	1	0	0	1	1	0	1	0	1	2	0	1	1	?	1	2	0	1	1	1	1	0	1	1	0	1	1	1	0	1	1	1	0	1	1	0	1	2	0	1	0	2	0	0	0	0	1	1	1	0	1	1	1	-	0	1	1	1	1	1
*Nautes magnificus*	0	1	0	1	2	0	1	0	1	2	1	0	1	1	1	1	2	0	1	0	1	0	0	0	0	1	0	0	0	0	0	1	1	1	1	0	1	1	1	0	1	1	0	0	1	0	1	0	2	0	0	0	0	1	1	1	0	1	1	0	1	0	1	1	1	1	1
*Tarpela depressa*	0	1	0	0	?	0	1	1	1	2	0	1	1	1	0	1	2	0	1	1	0	0	0	0	1	1	1	1	0	1	1	0	1	1	1	0	1	1	1	0	1	1	1	0	0	0	1	0	2	0	-	0	0	0	1	1	0	1	0	1	-	1	0	1	1	1	1
*Nautes varians*	0	0	0	1	0	0	1	1	0	1	0	1	0	1	0	1	2	0	0	0	1	0	0	0	1	1	1	1	0	0	-	0	1	1	1	0	1	1	0	0	1	1	0	0	1	0	1	0	2	0	0	0	0	1	1	1	1	1	1	0	1	0	1	1	1	0	1
*Tarpela contigua*	0	0	0	1	2	0	2	1	0	1	0	1	1	1	0	1	0	1	0	1	1	1	1	1	1	1	1	1	1	1	1	2	1	1	1	0	1	?	0	0	1	1	1	0	0	0	1	0	2	0	0	0	0	0	1	1	0	1	0	1	-	1	0	1	1	1	1
*Nautes belti*	0	1	0	1	2	0	2	1	1	2	0	1	1	1	0	1	2	0	0	1	1	0	1	0	1	1	0	0	0	1	0	0	1	1	0	0	1	1	1	0	1	1	0	0	2	1	1	0	2	0	0	0	0	1	1	1	0	1	1	0	1	0	1	1	1	1	1
*Nautes fervidus*	0	1	0	1	2	0	?	1	2	2	1	0	0	1	0	1	2	0	1	0	0	0	1	0	1	1	0	0	0	0	0	0	1	1	1	1	1	1	1	0	1	1	0	1	-	-	1	0	2	0	0	0	0	1	1	1	0	1	1	0	1	0	1	1	1	1	1
*Tarpela aerifera*	0	1	0	0	2	0	2	1	1	2	0	1	1	0	0	0	2	0	0	1	1	1	2	0	1	1	1	1	0	1	0	0	1	1	1	0	1	1	1	0	1	1	0	1	-	0	1	0	2	0	0	1	0	1	1	1	0	1	1	0	1	0	1	1	1	0	1
*Tarpela reticulata*	1	1	0	1	2	0	1	0	0	0	0	0	1	0	1	0	2	0	0	1	?	1	2	0	1	1	1	0	0	1	1	0	1	1	1	0	1	1	0	0	1	1	0	0	-	0	1	0	2	0	0	0	0	1	0	1	0	1	1	1	-	0	1	1	1	1	1
*Helops rufipes*	1	1	0	0	0	1	1	1	0	1	0	1	2	2	3	1	0	1	0	1	0	1	2	1	0	-	1	1	1	1	1	1	0	1	1	0	0	0	1	0	0	0	-	0	2	0	1	0	1	0	0	0	0	0	1	1	0	1	1	0	1	1	1	1	1	1	0
*Helops inanis*	0	1	0	0	2	0	0	1	0	2	0	1	1	1	0	1	0	1	0	1	1	1	1	1	0	-	1	1	1	1	1	1	1	0	1	0	1	1	1	0	1	1	0	0	1	0	1	0	2	0	0	0	0	1	1	1	1	1	0	1	-	1	1	1	1	1	1
*Tarpela costata*	0	1	0	0	0	0	1	0	0	0	0	1	1	0	1	1	2	0	0	0	0	1	2	0	1	1	0	0	1	1	1	2	1	1	1	0	1	1	0	0	1	1	0	0	1	0	1	0	2	0	0	0	0	1	1	1	0	1	1	1	-	0	1	1	1	0	1
*Nalassus plebejus*	1	1	0	0	0	0	1	1	1	2	0	1	0	1	0	1	2	0	0	1	1	0	0	0	1	0	1	1	0	1	1	2	1	0	0	0	1	1	1	1	0	1	-	0	1	0	0	0	1	1	0	1	0	0	1	1	1	-	0	1	-	1	1	0	1	0	0
*Helops aereus*	1	1	0	0	0	0	1	1	0	2	0	1	0	1	0	1	2	1	0	1	1	1	0	0	0	-	1	1	0	1	1	0	1	0	0	0	1	1	1	1	0	1	-	0	2	0	0	0	1	1	1	1	0	0	1	1	1	-	0	1	-	1	1	0	1	0	0
*Odocnemis californicus*	1	1	0	0	2	0	1	1	0	2	0	1	1	1	0	1	0	1	0	1	1	0	1	0	1	0	1	1	0	1	1	0	1	0	0	0	1	1	1	0	0	1	-	0	2	0	1	0	1	1	1	1	0	0	1	1	1	-	0	1	-	1	1	0	1	0	0
*Stenomax aeneus*	1	1	0	0	2	0	2	1	0	1	0	1	1	1	0	1	0	1	0	2	0	1	1	0	0	1	1	1	1	0	0	1	1	0	0	0	1	1	1	0	0	1	-	0	0	1	1	0	1	2	0	1	0	0	0	0	1	-	0	1	0	1	1	0	0	0	0
*Tarpela cordicollis*	1	1	0	0	2	0	2	0	1	2	0	1	1	1	0	1	0	1	0	1	1	0	1	0	1	0	1	1	0	1	1	2	1	1	0	0	1	1	1	0	0	1	-	0	2	0	0	0	1	1	1	1	1	0	1	1	1	-	0	1	1	1	1	0	1	0	0
*Hypogena biimpressa*	1	0	1	0	2	1	2	1	1	0	2	0	0	1	0	1	2	1	0	1	0	0	1	0	1	0	1	1	1	1	1	2	1	1	1	1	1	0	2	0	0	1	-	1	-	-	1	1	-	2	1	1	0	0	1	0	1	-	0	1	-	0	1	1	0	2	2
*Tenebrio molitor*	0	0	1	1	0	1	0	1	1	2	1	1	1	2	0	1	2	1	1	1	1	0	0	1	1	1	1	0	1	1	1	2	0	0	1	0	0	1	3	0	0	0	-	1	-	-	1	0	0	0	0	0	1	0	0	1	1	-	0	1	-	0	1	1	1	0	0

The consensus shows that the monophyly of the tribe Helopini is supported by one synapomorphy: the filiform antennae (3:0). In contrast, none of the subordinated taxa within Helopini is supported as monophyletic: neither the subtribes (Cylindrinotina and Helopina) nor the genera represented by more than one species: *Helops*, *Nautes*, or *Tarpela*. Cylindrinotina is nested within Helopina and *Tarpela cordicollis* (Marseul, 1824) plus *Helops aereus* Germar, 1824 (Helopina) are in turn nested within Cylindrinotina. *Helops* and *Tarpela* are polyphyletic, while *Nautes* is paraphyletic (*Helops farctus* LeConte, 1858, at some point transferred to *Nautes*, and *Tarpela aerifera* Allard, 1876 share a common ancestor with it).

From the sampled Palearctic Helopina, only *Helops rossii* Germar, 1817 and *Helops insignis* Lucas, 1846 constitute a clade that is sister to the rest of the tribe, and is supported by the pubescent ventrites with homogeneous punctures (32:1).

An heterogeneous clade formed by three genera of Cylindrinotina, *Stenomax aeneus*, Scopoli, 1763, *Odocnemis californicus* (Mannerheim, 1843) and *Nalassus plebejus* Küster, 1850 plus two species of Helopina: *Tarpela cordicollis* and *Helops aereus* is supported by the following internal characters: evident arms of the spiculum ventrale (34:0), dilated distal end of stalk of the spiculum ventrale (35:0), parameres without evident setae (57:1), and parameres with a keel (64:0), the last recovered as a synapomorphy. *Helops aeneus* was placed in *Stenomax* by Allard (1876) before Champion’s synonymization.

A large clade of mostly Neotropical species from the genera *Helops*, *Nautes* and *Tarpela*, plus two Nearctic species of *Helops*, was recovered with support from three characters: clypeus slightly depressed (1:0), more than one spermathecal tube (41:1) (retrieved as synapomorphy), and an accessory gland terminal to the spermathecal tubes (49:2) (retrieved as synapomorphy). *Helops punctipennis* LeConte, 1870 and *Helops rufipes* (LeConte, 1851), both Neartic, are supported as sister to this mostly Neotropical clade by two internal characters: the deep notch of the eighth sternite (52:0) and the lobate shape of the median lobe (66:1). The earlier divergent lineage within this mostly Neotropical species is a clade formed by *Tarpela contigua* Champion, 1887 and *Helops perforatus* Horn, 1880, supported by two internal characters: paraproct three or more times longer than the coxite (39:0) and the presence of a ventral projection at the parameres apex (63:0). *Helops inanis* Allard, 1877 and *Tarpela depressa* Champion, 1887 form a grade with respect to the remaining mostly Neotropical species. There is then a polytomy that includes *Tarpela torrida* Champion 1887, a clade with three *Tarpela* species including the type (*Tarpela browni* Bates, 1870), and another clade that is mostly composed of *Nautes* species and includes all the sampled species of this genus, even the type (*Nautes fervidus* Pascoe, 1866). The internal clade containing the type species of *Tarpela* also includes *Tarpela reticulata* Champion 1887 and *Tarpela costata* Champion 1887, and is supported by three characters of the pronotum: the gibbous surface of the disk (8:0), the crenate carinae of the lateral sides (14:0), and the acute anterior angles (15:1), plus one internal female character: the relative length of the paraproct (39:0). The mostly *Nautes* clade also includes *Tarpela aerifera* and *Helops farctus*. This group is supported by the short size of the fourth tarsomere (31:0) and the constriction of the apex of the parameres (60:0).

## Discussion

### Taxonomic implications

Although supported by our results, the monophyly of the tribe still requires a more rigorous test including a wider sample of species from more tribes including species from other closely related tribes (e.g. Triboliini, Blaptini). The only synapomorphy supporting the tribe, the filiform shape of the antennae, could be an artifact of our sampling, as the antenna have also been reported as moniliform or gradually clavate within the tribe (Aalbu et al. 2002). Based on our examination of many additional species, we know of no Helopini with moniliform or submoniliform antennae, nevertheless gradually clavate antennae are present in some species, such as *Nautes antennatus* Champion, 1887, *Nautes varians* Champion, 1887, *Helops durangoensis* Champion, 1887, and *Helops rufipes*.

The fact that Cylindrinotina is nested within Helopina implies that there is no justification for the recognition of two subtribes: either no subtribes should be recognized or more subtribes should be recognized. A denser sampling of Palearctic species could help reveal which of these alternatives is better supported. According to the current sampling, it is possible that the Palearctic *Helops* remain as an independent earlier divergent lineage within the tribe, including the type species (*Helops caeruleus*), which is morphologically similar to the sampled Paleartic species. If this was the case, *Helops* would have to be re-circumscribed to include only the Palearctic species and new generic names would be necessary for the New World lineages.

Further earlier divergent lineages may be revealed as sampling of *Entomogonus*, *Raiboscelis* and *Probaticus* is improved, as well as other genera not included in our sampling (e.g. *Catomus* Allard, 1876, *Hedyphanes* Fischer von Waldheim, 1820, and *Nesotes* Allard, 1876). The unresolved position of *Helops cisteloides* Germar, 1824 indicates the possibility that other New World lineages could be identified as sampling is increased. If subtribes are to be recognized, Cylindrinotina would need to be expanded to include Asian species of *Tarpela* (as *Tarpela cordicollis*) and Neartic *Helops* (as *Helops aereus*). This subtribe would also have to include several Holartic genera (besides *Odocnemis*). The Holarctic region has an intricate history (Brown and Lomolino 1998), with dispersion of groups taking place in several moments of the Tertiary (Sanmartín et al. 2001). The geographic heterogeneity of the cylindrinotine clade shows the importance of using a phylogenetic approach in which the morphological diversity of the taxa is represented, regardless of their present geographic distribution.

The polyphyletic nature of *Helops* and *Tarpela* render Champion’s classification (1887, 1893) and those of previous authors like Horn (1870) artificial. In contrast to Champion’s conservative classification, Allard’s classification (1876, 1877) was more natural in the sense that he recognized several lineages in the New World, some of them with Holarctic distribution. Allard’s placement of *Helops aereus* in cylindrinotine is supported by our results; nevertheless our results suggest that it should be classified in *Nalassus*, not in *Stenomax*. However, further analyses including more genera from the subtribe are necessary before taxonomic changes are made. This is also the case of the Asiatic *Tarpela cordicollis*, which was classified in a different sub-genus (*Lamperos*) by Allard (1877). Allard (1876) proposed the genus *Lamperos* to comprise some *Tarpela* species from North America and Japan, but later reduced it to subgenus (Allard, 1877), including *Tarpela cordicollis*. Aside from this species of *Tarpela*, all the others, including the type (*Tarpela browni*) are placed in a different lineage formed mostly by Neotropical species. This lineage, nevertheless, also includes species of *Nautes*.

The paraphyletic nature of *Tarpela* with respect to *Nautes*, could imply different outcomes as a wider taxon sampling (including more Nearctic species of *Helops*, *Nautes* and mainly *Tarpela*) and character (e.g. from DNA or fine structures revealed using SEM) is considered. Either several lineages could be recognized as different genera or all the species could be lumped in a single larger genus (*Nautes* due to nomenclatural priority, or if applicable, a conserved name *Tarpela*). Even if *Nautes* was supported as a different genus, taxonomic rearrangements seem to be likely. According to the current sampling, *Helops farctus* and *Tarpela aerifera* would need to be reassigned to *Nautes*.

### Morphology

Female genitalia have been used as a source of characters to study the relationships among suprageneric taxa in Tenebrionidae (Tschinkel and Doyen 1980, Doyen and Tschinkel 1982, Doyen 1994). Nabozhenko (2006) recognized four morphological patterns for the female genitalia that he associated to lineages from Helopina and Cylindrinotina, two patterns within each subtribe. In our sampling we only observed two of these patterns (Fig. 4A, B), but we also observed two patterns not previously reported for the tribe (Fig. 4C, D). Nevertheless, one of these patterns (Fig. 4C) was previously described for species belonging to Pimeliinae (Doyen 1994). These two patterns newly reported for Tenebrioninae were only seen in the mostly Neotropical clade. Most of the members of this clade share the pattern previously reported for Pimeliinae (Fig. 4C) and the pattern that we report here for the first time (Fig. 4D) was present only in the earlier divergent group of this clade (*Helops perforatus*-*Tarpela contigua*) as well as in *Tarpela depressa*.

Nabozhenko (2001b, 2002a, 2002b, 2005) describes the morphological patterns for the female genitalia tubes of the helopiod type as follows: basal spermathecal duct distinct; spermatheca consisting of two ducts of different length, without additional reservoirs and short processes; basal duct about as long as duct between place of running of gland and branching of spermatheca (Fig. 4A). The female genital tubes of the nalassoid type consist of a short and simple spermatheca, without lateral processes, reservoirs, and branching; gland short, about as long as spermatheca (Nabozhenko 2001b, 2002a, 2002b, Fig. 4B). The pattern shared with some Pimeliinae (Doyen 1994) consists of several spermathecal tubes close to each other or united at the base as a fascicle, always originating near or at the vagina apex, hence without a basal spermathecal duct (Fig. 4C). The newly documented pattern presents several spermathecal tubes distant from each other (Fig. 4D). In both cases, the accessory gland emerges from the common duct (if it is present), always in a terminal position with respect to the spermathecal tubes (Doyen 1994).

Due to its high variation, male genitalia have also been used to explore the relationships among species and higher taxonomic groups (e.g. Doyen and Tschinkel 1982, Aalbu 2005). As in the case of the female genitalia, Nabozhenko (2006) also recognized four morphological patterns for the male genitalia in lineages of the subtribes Helopina and Cylindrinotina. In contrast to the female genitalia, the morphological patterns found among the sampled species fit three of the previously described patterns by Nabozhenko (2006), only with what we consider a minor variation in the catomoid type. The patterns that we recognize correspond to Nabozhenko’s helopioid, nalassoid and catomoid types. According to Nabozhenko (2001b, 2002a, 2005) the helopiod male genitalia type in the broad sense (Fig. 5B) has, among other characters: heavily sclerotized parameres, covered with elongate punctures; baculiform sclerites of spiculum gastrale approximate, not curved outwards in dorsal view. The nalassoid male genitalia type (Figs 5A, E, and 6C, F) is characterized by: an aedeagus weakly sclerotized, semitransparent; parameres elongate, produced apically into compressed keel (Nabozhenko 2001b, 2002a). The catomoid male genitalia type is only present in the mostly Neotropical clade and is characterized by: penis with two or three apices, rounded in apical part; phallobase very long in comparison with short parameres; parameres with elongate aspirate punctuation and inconspicuous short hairs (Fig. 6B, E) (Nabozhenko 2006). The variation we found for all the species with respect to the catomoid aedeagus type is a lobate apical part of the penis and a shorter basal piece (relative to the length of the parameres) (Fig. 5F).

The recognition of the female and male genitalia types is translated into several homology hypotheses reflected in the matrix as characters 33 to 67 and their corresponding character states (see the list of phylogenetic data: characters above).

Although widely used as a taxonomic character, the keel on the parameres (64) has been reported as not always present through the subtribe (Nabozhenko 2001a). Nevertheless, this could be an artifact of the observation tools, as small keels can be detected when using a scanning electron microscope (SEM) (results not shown). For this reason we prefer to code this condition as “not evident” (see character 64) (in contrast to lacking). This is the same for the “absence” of setae on the parameres, here coded as “not evident” (see character 57).

Other diagnostic or traditionally used characters of the clypeus, antennae, prosternum, wings and tarsi were homoplastic but generally informative, contributing to the overall resolution of the tree. Only four characters from the original matrix introduced high levels of conflict, resulting in a lack of resolution in the consensus. These characters were all continuous and without a more refine codification, e.g., using statistical or morphometric tools, they only obscured the relationships posed by the remaining characters. On the other extreme, the shape of the antenna, generally considered to be a homoplastic character, was recovered as synapomorphic for the tribe. However, this synapomorphy needs to be tested with a broader taxon sampling.

## Conclusions

Although supported by our results, the monophyly of the tribe still requires a more rigorous test in terms of the taxon sampling from related tribes.

None of the subtribes or the analyzed subordinate genera of Helopini sampled by more than one species was corroborated as monophyletic. A wider taxon sampling is required to circumscribe them in a natural way.

*Helops* and *Tarpela* are polyphyletic, while *Nautes* is paraphyletic, and hence it is expected that further taxon and character sampling in a cladistic context will provide evidence for further splitting of *Helops* and *Tarpela* and a re-circumscription of *Nautes* including some *Helops* and *Tarpela*.

Our results show that in order to achieve a natural classification of Helopini, sampling of taxa should not be based on geographic distribution, although there might be some geographically correlated lineages. This approach has shown that there is a derived New World clade that is mainly composed by Neotropical species. Future efforts should also concentrate on increased sampling within this clade, to reveal other lineages or to corroborate the current ones, so that taxonomic changes can be concordantly proposed.

## Acknowledgments

The authors would like to thank the steering committee of the Third International Tenebrionoidea Symposium for the invitation to present our results, especially to Dr Aaron Smith, for his help and useful comments. We are also grateful to the curators of the collections mentioned in the materials and methods section for loaning the specimens used in this work. We greatly appreciate the revision and valuable comments by Dr E. Nearns and Dr M. Zurita-García, as well as those by two anonymous reviewers and most particularly to the associate editor, Dr Patrice Bouchard, who not only provided relevant comments, but was also very understanding and helpful during the publication procedure. We would like to thank S. Guzmán (IBUNAM) for her help with the use of the Leica equipment. The first author thanks the Posgrado en Ciencias Biológicas, UNAM, for its support and the fund of the “Programa de Apoyo para Estudios de Posgrado” provided to visit the entomology collections at the Natural History Museum (BMNH) and the Muséum National d’Histoire Naturelle (MNHN). This study was funded with a doctoral fellowship from the Consejo Nacional de Ciencia y Tecnología (CONACYT 202666) to the first author.
